# Ethical and technical considerations for the creation of cell lines in the head & neck and tissue harvesting for research and drug development (Part II): Ethical aspects of obtaining tissue specimens

**DOI:** 10.1186/1755-7682-2-9

**Published:** 2009-04-03

**Authors:** Tahwinder Upile, Waseem Jerjes, Panagiotis Kafas, Sandeep U Singh, Jaspal Mahil, Ann Sandison, Colin Hopper, Holger Sudhoff

**Affiliations:** 1 Head & Neck Centre, University College London Hospital, London, UK; 2 Head and Neck Department, Charing Cross Hospital, London, UK; 3 Department of Surgery, University College London Medical School, London, UK; 4 Department of Oral Surgery and Radiology, School of Dentistry, Aristotle University, Thessaloniki, Greece; 5 Department of Histopathology, Imperial College & Charing Cross Hospital, London, UK; 6 Department of Otolaryngology, Bielefeld University, Bielefeld, Germany

## Abstract

**Background:**

Although much has been published for the development of cell lines, these were lab based and developed for scientific technical staff.

**Objective of review:**

We discuss the ethical implications of tissue retention and present a generic consent form (Part II). We also present a simple and successful protocol for the development of cell lines and tissue harvesting for the clinical scientist (Part I).

**Conclusion:**

Consent is also more proximate and assurance can be given of appropriate usage. Ethical questions concerning tissue ownership are in many institutions raised during the current consenting procedure. We provide a robust ethical framework, based on the current legislation, which allows clinicians to be directly involved in cell and tissue harvesting.

## Background

In this molecular diagnostic age, we have a duty to our patients to try to advance and improve treatment. One of the main areas of research nowadays is related mainly to cell cultures [1-7] and their applications increases everyday.

Advances are dependent upon and limited by the availability of sufficiently high quality tissue samples for analysis by DNA, mRNA and expressed protein assays [8-10]. The limitations may be caused by restrictions in the scope of patient consent [11], rarity of the disease, diversity of tumour types, method of storage and inadequate documentation [9,10,12].

Although much has been published for the development of cell lines [1-7], these were lab based and developed for scientific technical staff. We, however, present a simple and successful protocol for the development of cell lines and tissue harvesting for the clinical scientist (see Part I). Our aim is to enhance the quality of translational research to the benefit of our future patients.

In this section (Part II) we discuss the ethics implications of tissue retention and present a generic consent form, which maybe adapted to suit individual institutions. The Human Tissue Act 2004 [11,13,14] provides a detailed statutory framework for tissue use but does not resolve the issues of ownership and of how much information should be disclosed to those consenting and how specific the consent should be [11], we hope to provide a framework within which this legalisation could be ethically adhered to.

## Methods

The Human Tissue Act 2004 [13] and Human Tissue Act 2006 (Scotland) [14] were passed following inquiries into the storage of children's organs and tissue without the proper consent. The Acts make consent [15-17] central to the lawful storage and use of children and young people's organs and tissue, and to the removal of such material after death. The Human Tissue Authority [14] regulates and issues codes of practice on activities covered by the Act in England, Wales and Northern Ireland. Scottish ministers have those powers in Scotland [15,18].

The taking of fully informed consent is more complicated than is commonly realised [11,13-15,17-21], (Appendix 1). Many of the previous guidelines involved obtaining a waiver from the patient at the time of consent for the potential commercial exploitation of their tissues by third parties with no direct personal gain for the donor. This may be akin to the exchange of precious artefacts for shiny beads; a familiar and often employed historical practice by which many of the early European and American settlers 'duped' native populations. This waiver may be worth in the order of many billions in currency and has now been successfully legal contested. As clinicians we have to be tirelessly cautious and honest to act in our patients 'true' best interests and not just be the confidence inspiring face that in times of personal crisis for of the vulnerable patient gets them to sign away their rights. An ethical approach however does not preclude considerable pharmaceutical profits or future investment in research and development, despite what we are commonly led to believe even as shareholders. Furthermore, complicity if proven may result in erase by our licensing bodies, since we may not have acted in our patients' best interests [13,15,18].

Consent for removal of tumour tissue during a surgical procedure should be distinct from consent for the retention of the tissues for future use in research for specified uses [22,23]. Two contrasting and non-coherent common law principles apply to the consent for surgery to remove samples and the new legislation regarding the storage and use of samples.

The consent to the procedure must be obtained from the person who has decision making capacity or in some cases parental responsibility if the test of capacity (Gillick) [9,16] is not passed although good practice would indicate that all parties involved should be in agreement (Appendix 1). Explaining the complex relationships between patient care, research and commercial biotechnology to the patient and their family during the consenting process to a surgical procedure with its attendant practical and emotional difficulties could be particularly challenging [9,18,22,23].

The needs for treatment have priority over the needs for research samples [23]. Individual clinical practices of multiple biopsies to reduce sampling errors and procedure repetition may lead to excess tissue but this cannot be guaranteed for research [9,10,23].

Tissue banking is of importance where the studies of tumours of low incidence (i.e. head and neck tumours) restrict the potential for large collections of tissues in small centres [24,25]. The types of samples collected would include solid tumours, normal tissue adjacent to the tumour, biopsies blood and bone marrow aspirates as well as extracted nucleic acids [25].

Many challenges will be faced by those running tissue banks this includes the need to respond to the ever changing law regarding this domain [13,15,22,24,25].

Complications arise because samples stored for research may remain after treatment is completed. Despite this it could be argued that the sizes of samples taken for research are small and there is no apparent current risk to the patient from the storage of tissues for research.

Unfortunately, with the advances in technology the idea that a tissue sample is really made anonymous by simple removal of patient details is now fallacious [25]. This is because with DNA fingerprinting all samples can now potentially be identified. Should this data be inviolate even against the vagaries of the law which may later dictate the sharing of all databases? There would be further implications for storage of paediatric tissue [22,23]. Once again just because sharing of tissue data is made legal this does not mean it is morally right or ethical.

The possibility of misuse imposes a responsibility of proper management and protection to the subjects' interests. Informed consent is required for all types of DNA banking. Objective ethical committee oversight is required to ensure an acceptable balance between risks and benefits [24,25].

## Results

Consent may differ for new and existing collections.

a) For **new collections **consent should be written and specific protection should be provided for vulnerable subjects, populations based on the principles of acting in their best interests. Individuals are informed of the types of research that will or might be carried out, whom should have access and the duration of storage. Consent should be freely given and be free of pressure and based on the validated information given by trained staff.

b) For **existing collections**, knowing that new technology allows patient identification from just DNA samples and that no true anonymisation can exist, the issues become more pertinent. Investigators should be obligated to re-contact subjects to obtain consent for new studies. Where impracticable new tissues should be sought [25]. Tissues should not be bartered for treatment which should be considered a proxy for the illegal organ trade. Post-mortem specimens should not be used if no consent was obtained. Commercial banks should be regulated and severely censured if they infringe agreed stipulations [9,12,14].

While protecting confidentiality, the free circulation and availability of genetic information and samples for research should be promoted [9,12,23].

The underlying principles which should apply:-

a) The subject should always be considered as the primary controlled of its DNA and clinical information directly derived from it. Once processed this information becomes research data with joint private ownership [24,25]. The clinician should be considered the patients advocate and custodian of the DNA/genetic data. As such they should take all appropriate steps to protect the data, its storage and access. It follows that the intellectual property should be that of the patient but with due consideration for benefit sharing. If a researcher or concern does not agree to these terms, market forces dictate that one that will agree may become available because of the 'precious' nature of the material [23].

b) For data already anonymised where it is not feasible to obtain patient consent, the material should be considered abandoned. It would be ethical to destroy the material and seek fresh samples.

c) The potential for future or actual financial exploitation of tissues for researchers/commerce creates an ethical imbalance for donor specific returns. A joint relationship obviates this inequity.

d) Use by third parties may be allowed provided there is no transfer in ownership of data or that derived.

### Ethics and consent

Research and ethical approval is ideally obtained with regards the consent and subsequent tissue use. The voluntary nature of the process must be emphasized and no form of duress implied, ideally the process is carried out well ahead of any procedure. We present our current consent form for modification and usage (Additional file 1).

Prospective patient data is entered on a proforma or directly into a database detailing i.e. family history, carcinogenic exposure, TNM stage (with volumetric staging), previous and proposed treatment and duration with later entry of prognostic, morbidity and mortality data. A note is made of the anonymised patient sample number. These anonymised records are held in a secure computer and written form.

## Discussion & Conclusion

We present a simple and successful protocol for the development of cell lines and tissue harvesting. It does not require high technology and can be performed by most clinicians in most hospitals. Rates of tissue registration at banks have been affected by the controversies surrounding the retention of human tissues and legal and ethical uncertainties [22,23]; we hope to provide an ethical and technical basis to reverse this trend.

The advantages of hospital based cell line creation are numerous. We can be more certain that cell lines are developed from the particular tissues of interest and accurate anatomical and appropriate clinico-pathological control tissues are also harvested.

Consent is also more proximate and assurance can be given of appropriate usage. Ethical questions concerning tissue ownership are in many institutions raised during the current consenting procedure [25]. Does the patient in his desire to cooperate with his doctor during cancer treatment really understand the ramifications of agreeing to the use of their tissues for research and potentially very lucrative drug development by third parties? Are they not put under undue influence? Do we as the clinicians directly dealing with the patient not have a duty of care to protect their interests rather than devolving responsibility to other organizations and committees who do not possess this overriding agenda [16,18].

The commercialisation of therapeutic products containing regenerative human tissue is regulated by the common law, statute and ethical guidelines in Australia and England, Wales and Northern Ireland. Brown and Then examined the regulatory regimes in these jurisdictions and considered whether reform is required to both support scientific research and ensure conformity with modern social views on medical research and the use of human tissue. The authors considered the crucial role of informed consent in striking the balance between the interests of researchers and the interests of the public [26].

Again, no current consenting procedure outlines this administrative area. When most patients consent for their tissues to be used, it is in direct reference to the doctor taking the consent and tissue. Perhaps as doctors, we should not be eager to overlook this point and perhaps use this to direct research into more clinically relevant areas to enhance translational utility. Problems further arise in the laboratory situation where such a precious resource is at a premium. When one operates there is a personal and professional trust between the patient and doctor, perhaps this should be extended to the research realm. Patients do not expect their tissues to be sold or offered to profit making organizations even if this is decided 'in-proxy' by tissue bank committee [24,25]. Should each separate use of patient's tissue require further direct patient consent? Despite these concerns, it is not difficult to imagine an equitable process whereby the use of patient samples are followed (traced) and if found valuable the patient have a direct share of the royalties from this. Similar to the entertainment industry and repeat performance royalty payments.

Currently when a tissue bank is created, it becomes an extremely valuable resource where many conflicting non-patient centred issues often occur. Even a tissue bank committee made up invariably of interested parties cannot truly be said to represent an individual patients view, (despite an often inclusion of a lay membership).

Morrin et al. reported the development of a central resource of consented cancer tissues for researchers to use for ethically approved projects. Most donors (99.6%) consented to allow access to medical records; 98.3% to tissue being sent overseas, 97.4% to commercial research and 35.6% requested disposal with a karakia. The predominant tissues are from donors with cancers of the breast, colon, urological, and gynaecological sites. they concluded that the tissue bank (in Christchurch) is a successful model, providing quality tissue samples for cancer research whilst appropriately addressing ethical, legal, and cultural aspects of their collection [8].

Philosophically, the agreement of an ethical approval committee does not necessarily mean that the act or process is ethical; examples exist where later evidence contradicts or overturns a local decision. Presently it is felt that selling or bartering of tissue is unethical even when carried out by non profit making organizations. Furthermore, for the consenting procedure to be full and ethical, it should represent an agreement of intent between the patient and his clinician only. The samples remain the non-transferable property of the patient only and their use directed by the harvesting doctor and pathologist after agreement with the patient. The samples should be used for a specific purpose, excess material is destroyed [11,17]. In research and drug development tissue samples are akin to 'gold-dust' and must be treated as such, i.e. not wasted for lack of taking and not abused. Any other scenario would welcome litigation. Public private partnerships still mean that any drug which is developed is still in license and the money comes from the public coffers. Lack of business insight on behalf of the scientists and clinicians should not be overcome by short term financial gain or inducements offered by commercial interests.

## Competing interests

The authors declare that they have no competing interests.

## Authors' contributions

TU, WJ, PK, SUS, JM, AS, CH, HS: contributed to conception and design, carried out the manuscript editing and manuscript review. All authors read and approved the final manuscript.

## Appendices

Appendix 1: Summary of relevant UK legislation

### Mental Capacity Act 2005: 5 Statutory Principles [18,20,21]

Subjects assumed to have capacity unless it is established that they lack the capacity

Subjects cannot be treated as unable to make a decision unless all practicable steps to help them do so have been taken without success.

Unwise decisions do not mean incapacity to make decisions

All decisions under the Act for a subject who lacks capacity must be done in their best interests

All decisions must be made in such a way as to be least restrictive to the patient and maximise the available their future choices.

### 'Gillick competence' for patient under 18 years old [17,22,23]

The subject has the maturity and intelligence to understand the nature and implications of treatment as assessed by the doctor.

The subject can consent to treatment if competent.

The subject may not be able to decline treatment if found to incompetent.

### Principles of Robust Consent [12,13,16-19,24]

Presume capacity and discuss risks, complications and benefits in an open and honest manner in a way designed to ensure patient understanding.

Ensure potential relevant future events and alternative actions are considered.

Provide options that would be least restrictive to the subject's future choices.

Ensure decisions are voluntary.

Respect the subject's decision and right to change their decision.

## Supplementary Material

Additional file 1 **Generic consent form.** Click here for file
