# Supplementary material for: Ethical and technical considerations for the creation of cell lines in the head & neck and tissue harvesting for research and drug development (Part II): Ethical aspects of obtaining tissue specimens
Source: Int Arch Med. 2009 Apr 3;2:9. doi: 10.1186/1755-7682-2-9 (PMC2670271; doi:10.1186/1755-7682-2-9)
Supplement: Additional file 1 — Generic consent form. [file 1755-7682-2-9-S1.doc]

**Department of Surgery** – Tissues for Research

Patient Details (or pre-printed label)

Patient’s surname/ family name

Patient’s first name

Date of Birth

NHS number:

Hospital Number

Male / Female

Special Requirements

(i.e. other languages/ other communication methods)

Doctor:

The **Department of Surgery** was founded to carry out research into the causes of diseases (i.e. cancer) and their treatments, and this remains one of its main aims. Tissue and /or blood samples are frequently very important for research and this form invites you to agree to assist us in various areas of our work only. Tissues in excess of diagnostic need which would usually be discarded will be used. Your treatment will in no way be affected. Your agreement is of course entirely voluntary and will not in any way affect your treatment.

Please write your initials by each section. You do not need to agree to all requests below.

**Section A; Research**

Consent for use and storage of tissues removed at surgery.

1. I agree to donate any excess tissue removed at surgery and blood samples to be used in future research projects, providing this does not in any way affect any diagnosis or treatment. I understand that this tissue and clinical information gathered about me can be stored by The **Department of Surgery** and our associated laboratories. The doctors involved with your clinical care will be the legal custodians of this material. I understand that tissue and clinical information can only be used in research projects if they have received approval of the Clinical Trustee’s and Ethics Committee. Tissue or its derivates will not be used without the permission of the Clinical Trustee’s. I also understand that some of these projects may involve researchers outside the **Department of Surgery** and our associated laboratories including workers in commercial companies. Any commercial use of donated tissue or its derivatives will be in favour of the patient and the **Department of Surgery** to improve ongoing patient care.

Notes to confirm patients understanding of:

2. I understand that my doctor and I will be informed if any results of the medical tests done as part of research are important for my health, where such results have not been anonymised

3. I understand that I will not immediately benefit financially if this research leads to the development of a new treatment or medical test. This agreement does not however imply I relinquish any future material benefit that may directly result from the use of my tissues.

4. I understand that I will be informed about how to contact the research team if I need to, and about how to get information about the results of the research.

**Section B: Quality Assurance and Education**

Consent to use medical records for quality assurance/ educational purposes.

5. I agree that information stored about me during any treatment at the **Department of Surgery** may be shared for quality assurance and/or education and may be shared with other Health and Research organisations.

**Section C: Genetic Research**

Consent to use samples for genetic studies

6. I agree that the samples I may give may also be used for genetic studies on the understanding that the samples will be anonymised (i.e. there will be no means of identifying me and no means of knowing the results of these tests).

Notes to confirm patient understanding

7. I understand that any future research on my samples involving genetics, which is not anonymised, will require separate consent from me.

If you wish to change your answers to any of the above at any time, please contact…

(Block Capitals)

Name of Patient: Date: Signature:

Name of person taking consent: Date: Signature:

Designation of person taking consent.
